# Valuation of health losses of women with multiple roles using a well-being valuation approach: Evidence from Japan

**DOI:** 10.1371/journal.pone.0251468

**Published:** 2021-05-12

**Authors:** Narimasa Kumagai

**Affiliations:** Faculty of Economics, Seinan Gakuin University, Fukuoka, Japan; University of Jyvaskyla, FINLAND

## Abstract

Unpaid housework among married working couples is largely done by women in Japan, causing health losses due to work-to-family conflict. However, monetary values for the poor health condition of working mothers with multiple roles have not been explored. The purpose of this study is to examine the impacts of health conditions on life satisfaction (LS) among middle-aged Japanese men and women and attach a monetary value to self-assessed poor health (SAPH). The well-being valuation approach applied monetary values to health losses among middle-aged working persons, using a total of 6,779 married workers drawn from a nationwide 6 wave (2007, 2009, 2011–2014) longitudinal data from the Japanese Life Course Panel Survey of Middle-aged Persons. Female workers having multiple roles as employees and housewives, who spent at least 35 hours per week on market work are defined as women with multiple roles. LS was used as a proxy of individuals’ subjective well-being. Considering the endogeneity between SAPH and LS, I used the two-stage residual inclusion approach with generalized residuals. Major findings are (1) health losses of women with multiple roles were 1.47 times of the equivalent household income; larger than those of men with multiple roles, and (2) health losses of women with multiple roles can be reduced by around 9.5% of the equivalent household income if the spouse shares the housework by engaging in frequent cleaning of the house. Taking health losses of women with multiple roles into consideration, middle-aged men should reconsider the allocation of work attributable to the attitudes toward gender roles.

## Introduction

Are middle-aged women who engage in several roles, healthier and happier than those with fewer roles? Research on the effects of multiple roles of employee, mother, and spouse on women’s health have found little evidence that the role combination of employment and motherhood resulted in harmful health effects [1–5]. Interestingly, the association between women’s employment status and their perceived health is not similar among European countries. Part-time employment was associated with better health than full-time employment among mothers in the Netherlands [6]. In contrast, full-time employment was associated with better health among women with a spouse and children in the four Nordic countries with a tradition of equal opportunity policies [7]. No difference in health between part-time and full-time employed mothers was found in Germany [8].

Conflicts between family and work roles appear to have detrimental effects on the health of working mothers. In a comparison of data from three countries (United Kingdom, Finland, and Japan), a study [9] showed that the poorer mental health of working Japanese women could be attributable, in part, to higher levels of work-to-family conflict (WFC). As Japan has been falling behind in promoting women’s health, researchers should assess the negative impacts of poor health conditions of working mothers with multiple roles in Japan. Furthermore, the difficult working conditions in Japan may explain the relatively poor health of the younger working women with family demands in their 30s [10].

The number of women with multiple roles has increased in Japan, but women still tend to assume primary responsibility for household duties, alongside their paid jobs. Most working Japanese women with multiple roles appear to face high levels of family-to-work or WFC because unpaid work among married working couples is largely done by women. According to the 2011 (2016) Survey on Time Use by the Statistics Bureau, Ministry of Internal Affairs and Communications in Japan, married working women spent an average of 3h27m (3h16m) a week on housework, and 45m (56m) on caring for children and other household members (total time spent on unpaid domestic and care work 4h53m, 4h54m). In contrast, married working men spent an average of 39m (46m) per week on household and family responsibilities.

Considering the disparity in the time spent on housework and childcare between married couples, Japanese researchers examined the life satisfaction (LS) of married people and revealed that a higher ratio of women’s income to household income was associated with low LS of married women [11]. However, no systematic relationships between multiple roles, health status, and LS were examined in the study; although women who occupied several roles in poor health might give their life satisfaction a low grade.

The well-being valuation approach applies monetary values to chronic diseases [12], 13 health conditions [13], and chronic pain [14]. As satisfaction is assumed to be less volatile and more cognitive-oriented than happiness [15–17], previous studies have estimated the income equivalent of health satisfaction changes using the subjective well-being (SWB) valuation approach and found that the main determinants of LS are health satisfaction [12, 18], health status, household income [13, 14, 19–22], age, gender [19, 20], employment status, and social relationships [20]. However, the impacts of poor health conditions of working mothers with multiple roles on their LS are still unclear. Thus, I used data of self-assessed health status and LS to estimate the monetary values for worker’s health loss. Since married and partnered mothers reported better mental health than single parents [4, 9], I focused on middle-aged married women who engaged in housework almost every day and spent at least 35 hours per week for market work (women with multiple roles). A recent study [23] showed that the husband’s contribution to housework had a significant positive effect on the wife’s labor participation. The results imply that changing the husband’s work style and his view on gender roles are effective in promoting the labor participation of married women.

Addressing the potential omitted variable bias is one of the statistical challenges in the study measuring health losses. This study copes with the endogeneity between household income, health, and LS to evaluate the health losses of women with multiple roles. The positive relationship between SWB and health holds well when using LS as the dependent variable in regressions [24–26]. Rojas [27] used a domains-of-life approach on seven areas including health, economic situation, and occupation, and focused on estimating the monetary values of illnesses such as cardiovascular disease or cancer. Causality seems to run in both directions: a high level of well-being is relevant for subsequent good health, but the stronger relationship seems to be between health and SWB [28].

Despite the uneven time allocation for family responsibilities, healthy-worker effects may exist among Japanese working mothers, where women with multiple roles are healthier and happier than working mothers with fewer roles. In contrast, role strains are harmful to health, where working mothers with fewer roles are healthier and happier than women with multiple roles. I compared the effects of health losses of women with multiple roles on LS vis-à-vis working mothers with few roles and provided results on specific conditions and their impacts on LS.

The rest of the paper is organized as follows. Section 2 presents the hypotheses to be tested and describes the variables of interest and the empirical methods. I used a nationwide 6 waves (2007, 2009, 2011–2014) longitudinal data set of the Japanese Life Course Panel Survey of the Middle-aged (JLPS-M). Section 3 reports the empirical results and discusses the specificity of this study. The results suggest that health losses of women with multiple roles can be reduced by around 9.5% of the equivalent household income if their husbands frequently engage in house cleaning activities. Finally, Section 4 offers the conclusions.

## Methods

### Data collection

For the empirical analysis, I used a nationwide 6 waves (2007, 2009, 2011–2014) longitudinal data set from the JLPS-M that focused on male and female Japanese residents aged between 35 and 40 at the time of the survey, which was conducted from January to March 2007. The respondents were selected from the Basic Resident Registration through a stratified sampling method based on age and gender. The data of the JLPS-M was provided by the Social Science Japan Data Archive, Center for Social Research and Data Archives, Institute of Social Science, The University of Tokyo. The data were analyzed anonymously. The JLPS-M in 2008 and 2010 does not provide information about the weekly housework frequency.

LS is assessed using the question, “How satisfied are you with your life?” and the responses are scored as “5” for totally satisfied and “1” for totally dissatisfied (I recoded the values of the original survey). The distribution of the responses to this question is slightly different for women and men. As Table 1 shows, the standard deviation of LS among women with multiple roles is 0.909, slightly lower than that of men with multiple roles. Each mean of LS is 3.810 for women with multiple roles and 3.814 for men with multiple roles. The coefficient of variation among women with multiple roles is 0.239, slightly higher than 0.237 for men with multiple roles. The prevalence of self-assessed poor health (SAPH) among women with multiple roles is 0.155, lower than 0.154 of men with multiple roles. The gender difference in LS and SAPH among middle-aged workers with multiple roles is minor.

**Table 1:** Characteristics of selected variables in the middle-aged workers.

	Female Workers with Multiple Roles			Female Workers With Few Roles			Male Workers with Multiple Roles			Male Workers with Few Roles		
**Variables**	*N*	Mean	SD	*N*	Mean	SD	*N*	Mean	SD	*N*	Mean	SD
**Life satisfaction**	1,615	3.810	0.909	1,934	3.811	0.907	1,548	3.814	0.905	797	3.657	0.944
**Self-assessed health**	1,626	3.295	0.861	1,940	3.255	0.859	1,559	3.293	0.858	800	3.243	0.829
**Dummy variable for self-assessed poor health**	1,631	0.155	0.362	1,943	0.169	0.375	1,564	0.154	0.361	804	0.157	0.364
**Number of days for doctor’s consultation during the past year**	1,436	4.864	9.611	1,793	6.809	14.818	1,371	4.823	9.306	676	4.580	13.635
**Number of hospitalization days during the past year**	1,238	0.480	3.261	1,566	0.695	4.515	1,179	0.466	3.272	599	0.621	9.002
*Mental health inventory (MHI-5) (1 = all of the time*, *0 other)*												
**Being very nervous**	1,631	0.034	0.181	1,943	0.039	0.194	1,564	0.033	0.179	804	0.053	0.225
**Being depressed**	1,631	0.017	0.128	1,943	0.013	0.113	1,564	0.017	0.128	804	0.027	0.163
**Felt so down in the dumps that nothing could cheer you up**	1,631	0.015	0.123	1,943	0.015	0.121	1,564	0.015	0.123	804	0.021	0.144
**Felt calm and peaceful (1 = none of the time, 0 other)**	1,631	0.037	0.188	1,943	0.036	0.186	1,564	0.038	0.192	804	0.067	0.250
**Being happy (1 = none of the time, 0 other)**	1,631	0.013	0.113	1,943	0.019	0.137	1,564	0.013	0.115	804	0.029	0.167
**Equivalent household income (during the past year, logged)**	1,437	5.86	0.50	1,602	5.72	0.55	1,377	5.86	0.50	679	5.81	0.66
**Age**	1,631	42.77	2.68	1,943	43.13	2.61	1,564	42.72	2.69	804	42.44	2.74
**No of family members (*N*)**	1,617	3.93	1.37	1,925	4.14	1.38	1,550	3.91	1.37	798	3.90	1.46
**Dummy variable for hoping to be regular worker ten years later**	1,628	0.695	0.461	1,927	0.195	0.396	1,561	0.709	0.454	799	0.645	0.479
*Housework engaged at high frequency in the home*												
**Frequency of preparation for meals by the spouse**	1,492	4.570	1.990	1,854	1.699	1.243	1,425	4.714	1.907	712	5.524	1.083
**Frequency of washing by the spouse**	1,489	4.347	2.008	1,855	1.453	1.115	1,422	4.488	1.934	713	5.276	1.299
**Frequency of cleaning by the spouse**	1,488	3.638	1.838	1,856	1.575	0.953	1,422	3.728	1.817	712	4.379	1.524
**Frequency of shopping for meals by the spouse**	1,489	3.712	1.486	1,847	2.129	1.073	1,422	3.790	1.456	711	4.263	1.185
**Frequency of preparation for meals by self**	1,615	2.958	2.032	1,938	5.648	0.914	1,548	2.830	1.978	803	2.030	1.451
**Frequency of washing by self**	1,623	2.856	2.057	1,939	5.527	1.003	1,556	2.721	1.993	804	1.989	1.478
**Frequency of cleaning by self**	1,623	2.498	1.422	1,941	4.571	1.341	1,556	2.357	1.272	802	1.885	0.980
**Frequency of shopping for meals by self**	1,623	2.892	1.216	1,936	4.205	1.065	1,556	2.821	1.171	802	2.493	1.152

Sources: Japanese Life Course Panel Survey of Middle-aged Persons (JLPS-M) 2007, 2009, 2011–2014.

Respondents’ mental health was measured using the five-question Mental Health Inventory (MHI-5) as follows: “How much time during the last month have you: (i) been very nervous?; (ii) been depressed?; (iii) felt calm and peaceful?; (iv) felt so down in the dumps that nothing could cheer you up?; and (v) been happy? The respondents evaluated the frequency of each question as follows: all of the time = 1, most of the time = 2, some of the time = 3, a little of the time = 4, and none of the time = 5. For three variables (i, ii, iv), we inverted those values so that all of the time = 5, most of the time = 4, and so on until none of the time = 1. Hence, the highest score of “(v) been happy?” implies “none of the time = 5.” The prevalence score of nervous, depressed, and down in the dumps among male workers with few roles was the highest across samples in this study. This suggests that mentally healthy-worker effects may exist among Japanese working mothers with multiple roles, compared to male workers with few roles. Almost 70% of women with multiple roles and men with multiple roles did hope to be regular workers ten years later, but most female workers with few roles did not hope so. However, about 2.5% of women with multiple roles (*N* = 41) and men with multiple roles (*N* = 38) responded with the lowest score of LS, and they seemed to have a high prevalence score of poor mental health status, which was at most 11 times of mean prevalence (not shown). Working persons with multiple roles reported the lowest level of LS. This group might experience a substantial work-to-family conflict (WFC) because their prevalence of poor mental health status, such as feeling depressed, was high in women and men (17%, 16%).

Missing household income among married couples was almost missing at random (*N* = 342). No significant correlations between missing income and LS or self-assessed health were found among female workers; there was a small negative correlation between missing income and LS at the 5% significance level (ρ = -0.04).

### The well-being valuation approach

The objective of the econometric analysis is to investigate how an individual’s LS is affected by poor health status. An important issue in this analysis is that the main explanatory variable of interest, SAPH, is arguably not exogenous. When estimating an individual’s SAPH or LS, I took the effects of unobserved heterogeneity (e.g., measurement error, omitted variable bias, and reverse causation) into account. First, the instrumental variable method (IV probit) was used to correct probable bias in the estimated parameters measuring the relationship between SAPH and the natural logarithm of equivalent household income (EHI) [19]. The standard transformation to natural logarithms was done to account for the diminishing marginal effect of income on health. Second, LS might be significantly correlated with certain omitted variables regarding SAPH because poor health is a predictor of life dissatisfaction but the reverse causal relationship has not been established [29]. To provide an accurate correction for small amounts of endogeneity, following the procedure of the two-stage residual inclusion (2SRI) approach [30], I added the generalized residual of the reduced-form equation to solve the endogeneity problem in discrete models.

To measure the lost value of moving from good/very good/excellent health to fair/poor health, I used the dichotomous variable of SAPH.

$$SAPH_{i,t}=\beta_{0}+\beta_{1}X_{i,t}+\beta_{2}Y_{i,t}+\beta_{3}MH_{i,t}+\beta_{4}Z_{i,t}+\sigma_{i,t}$$ (1)

where X is the vector of demographic and socio-economic variables such as owing a house, Y is the real EHI adjusted for household size during the past year (base year = 2010), MH is the vector of mental health variables, Z is the instrumental variable, and σ is the error term. The subscripts *i* and *t* indicate the individuals and period, respectively. Hours worked per week at the previous survey were used as an IV. For workers with few roles, 20–30 hours in females and 35–40 hours in males were used, respectively.

A study by Brown [19] used a proxy for intelligence as an instrumental variable to measure the lost value of SAPH (moving from good/excellent health to fair/poor health), and reported monetary values of health loss in the long run for the U.S. population were US$41,654, which was 1.8 times median equivalized annual household income (2010 constant dollars). However, I selected the 2SRI approach because the JLPS-M did not provide the same data which can be used as a proxy for intelligence in large-size data.

The satisfaction model enables the estimation of monetary compensation for the losses in well-being caused by poor health. I estimated Eq 2 and derived monetary values of SAPH as follows (see [13, 20–22] for a more detailed exposition): (exp(mean of logged EHI during the past year)-1)×(exp(-γ_1_/γ_3_)-1).

Two hypotheses to be tested are (1) health losses of women with multiple roles were larger than those of men with multiple roles, and (2) health losses of women with multiple roles can be reduced if the spouse shares the housework.

The 2SRI approach with generalized residuals is supposed to produce the least bias when estimating the effect of the change in SAPH [31]. The disturbance distribution may change due to an omitted variable bias, which would lead to inconsistency in the method of estimation. To test H_0_: ρ_1_ = 0, I estimated a random-effects ordered probit regression using the residual of Eq (1) $\rho_{1}{\hat{\sigma }}_{i,t}$ as an explanatory variable of Eq (2).

$$LS_{i,t}=\gamma_{0}+\gamma_{1}SAPH_{i,t}+\gamma_{2}W_{i,t}+\gamma_{3}Y_{i,t}+\gamma_{4}H_{i,t}+\rho_{1}{\hat{\sigma }}_{i,t}+\varepsilon_{i,t}$$ (2)

where W is the vector of demographic and socio-economic variables used to model the SAPH, H is the vector of regional or city-size dummy variables, and ε is the error term. All models included frequency of spouses’ housework engagement in a week such as cleaning, preparation for meals, shopping for meals, and washing.

## Results

All the results of the weak instrument robust test (AR test) for IV probit models indicated that the null hypothesis of zero correlation coefficient between the error terms of SAPH and EHI was not rejected. All estimated models indicated zero correlation coefficient between the error terms (see, ath*rho* in Table 2).

**Table 2:** IV probit models.

	Female Workers with Multiple Roles		Female Workers with Few Roles		Male Workers with Multiple Roles		Male Workers with Few Roles	
Variables	Self-assessed poor health	Equivalent household income	Self-assessed poor health	Equivalent household income	Self-assessed poor health	Equivalent household income	Self-assessed poor health	Equivalent household income
**Equivalent household income (logged)**	0.254		-0.0423		0.545		-0.476	
	(0.937)		(1.547)		(0.557)		(0.920)	
**Age**	-0.0227	0.00957	0.0349	0.0217***	-0.0142	0.00982	-0.00389	0.0180*
	(0.0231)	(0.00637)	(0.0361)	(0.00608)	(0.0226)	(0.00640)	(0.0388)	(0.0102)
**Dummy variable for owning a house**	-0.376***	0.0880**	-0.102	0.0246	-0.404***	0.0767**	-0.266	-0.0590
	(0.133)	(0.0371)	(0.114)	(0.0348)	(0.122)	(0.0376)	(0.168)	(0.0541)
**Dummy variable for the high frequency of activities limited due to poor health**	omitted		1.943***	-0.182	omitted		omitted	
			(0.681)	(0.144)				
**Being very nervous**	0.707***	-0.0574	0.375	0.0834	0.724***	-0.0565	0.240	0.0247
	(0.268)	(0.0959)	(0.279)	(0.0851)	(0.272)	(0.0963)	(0.414)	(0.136)
**Being depressed**	0.0284	0.0424	0.583	0.290	-0.0579	0.0987	0.794	0.0423
	(0.570)	(0.203)	(0.826)	(0.256)	(0.572)	(0.201)	(0.620)	(0.237)
**Felt so down in the dumps that nothing could cheer you up**	0.629	-0.0919	-0.134	-0.279	0.703	-0.159	0.427	-0.515
	(0.588)	(0.208)	(0.832)	(0.243)	(0.588)	(0.204)	(1.066)	(0.352)
**Felt calm and peaceful (1 = none of the time, 0 other)**	0.533*	-0.00579	0.0843	0.0114	0.551**	-0.0212	-0.00683	-0.105
	(0.279)	(0.0945)	(0.273)	(0.0920)	(0.276)	(0.0929)	(0.404)	(0.121)
**Being happy (1 = none of the time, 0 other)**	0.247	-0.0393	0.388	-0.0825	0.277	-0.0549	-0.225	0.511**
	(0.444)	(0.155)	(0.432)	(0.151)	(0.439)	(0.153)	(1.082)	(0.223)
**Number of days for doctor’s consultation during the past year**	0.0296***	0.00213	0.0253***	0.000554	0.0360***	0.00151	0.0436***	0.00611*
	(0.00656)	(0.00190)	(0.00397)	(0.00103)	(0.00705)	(0.00210)	(0.0100)	(0.00368)
**Number of hospitalization days during the past year**	0.0420**	-0.00377	0.0290	-0.000616	0.0342**	-0.00285	0.121*	-0.0312*
	(0.0167)	(0.00509)	(0.0188)	(0.00419)	(0.0167)	(0.00516)	(0.0653)	(0.0173)
**Frequency of exercise in a daily life**	-0.147***	0.0439***	-0.0966	0.0388***	-0.156***	0.0422***	-0.0757	0.0714***
	(0.0533)	(0.0112)	(0.0747)	(0.0111)	(0.0463)	(0.0113)	(0.110)	(0.0187)
**IV: Dummy variable for 40 hours or more (20–30 hours / 35–40 hours) worked per week at the previous survey**		-0.138***		-0.0795**		0.181***		0.171***
		(0.0370)		(0.0392)		(0.0309)		(0.0500)
							
**Ath*rho***	-0.139		0.0276		-0.273		0.219	
	(0.462)		(0.793)		(0.288)		(0.510)	
**lnσ**^**2**^	-0.733***		-0.671***		-0.750***		-0.652***	
	(0.0221)		(0.0202)		(0.0227)		(0.0320)	
**AR test: chi**^**2**^**(1), Prob > chi**^**2**^	0.07 (0.78)		0.00 (0.97)		0.85 (0.35)		0.24 (0.62)	
***N***	1,022	1,022	1,221	1,221	967	967	487	487

Note: Changes in LS, frequency of 3 times eating a day, and constant term were included.

Standard errors in parentheses *** p<0.01, ** p<0.05, * p<0.1.

The results of second-stage regression among women with multiple roles and men with multiple roles showed that being very nervous had positive effects on SAPH at the 1% significance level. The number of hospitalization days during the past year also had positive effects on SAPH at the 5% significance level. While Sumra and Schillaci [32] found a significant negative relationship between the number of roles and exercise frequency, women and men with multiple roles, who had the habit of exercising daily did not tend to assess subjective poor health status. The number of days spent for doctors’ consultations during the past year was positively associated with SAPH across all samples.

Generalized residuals were not significant at the 10% level among women with multiple roles or men with multiple roles, and then random-effects ordered probit regression gave monetary values to health losses for women and men with multiple roles (Table 3). In contrast, generalized residuals were positively significant at the 1% level among working mothers or fathers with few roles, and thus the endogeneity bias remained. Here, we cannot obtain accurate monetary values for their health losses.

**Table 3:** Random-effects ordered probit models: 2SRI approach.

Dependent variables: LS	Female Workers with Multiple Roles	Female Workers with Few Roles	Male Workers with Multiple Roles	Male Workers with Few Roles
**Self-assessed poor health**	-0.954**	-1.962***	-0.856*	-2.960***
	(0.382)	(0.514)	(0.450)	(0.718)
**Generalized residual**	0.287	0.769***	0.204	1.311***
	(0.230)	(0.261)	(0.250)	(0.366)
**Equivalent household income (logged)**	0.649***	0.477***	0.594***	0.315*
	(0.187)	(0.110)	(0.174)	(0.178)
**Age**	-0.00123	-0.0697	0.0117	0.0369
	(0.0472)	(0.0494)	(0.0699)	(0.0833)
**Dummy variable for owning a house**	0.433**	0.360*	0.481**	0.161
	(0.188)	(0.192)	(0.200)	(0.285)
**Dummy variable for income ratio to household income (1 = 50% or more, 0 other)**	0.743**	-0.0606	0.718**	-0.590
	(0.365)	(0.516)	(0.363)	(0.904)
**Frequency of preparation for meals by the spouse**	-0.0828	-0.146**	-0.0628	0.0678
	(0.0629)	(0.0739)	(0.0739)	(0.140)
**Frequency of washing by the spouse**	-0.0209	0.0302	-0.0274	0.0122
	(0.0659)	(0.0739)	(0.0712)	(0.105)
**Frequency of cleaning by spouse**	0.187***	0.0619	0.186***	0.0706
	(0.0675)	(0.0925)	(0.0624)	(0.0723)
**Frequency of shopping for meals by the spouse**	-0.0373	0.169**	-0.0361	-0.0967
	(0.0669)	(0.0708)	(0.0668)	(0.0823)
**lnσ**^**2**^	2.043***	1.949***	2.098***	1.254***
	(0.418)	(0.248)	(0.396)	(0.337)
***N***	1,007	1,202	957	476
**Average *N* per samples**	433	420	407	224

Note: Regional dummy variables such as Hokkaido, city size, frequency of preparation for meals by myself, frequent conversation with friends, and year dummy variable were included.

Standard errors in parentheses *** p<0.01, ** p<0.05, * p<0.1.

Surprisingly, a higher ratio of income of women with multiple roles to household income was positively associated with LS at the 5% significant level. However, the estimation results showed the monetary values for health losses of women with multiple roles were 1.47 times (= 0.954/0.649) of the annual EHI (US$32589.88 = JP¥3328.8 thousand ×1.028, 105 /US$, 2010 constant dollars), larger than those of men with multiple roles. This annual monetary value was derived from the EHI of workers’ households (4 persons per household) in 2014, and about 78.2% of US$41654 [19]. Because the worker’s household income has only slightly increased during the long-term economic stagnation in Japan, we consider that the amount of monetary values of health losses were below the estimates for the U.S. population. The employers in a society where the number of the middle-aged labor force has declined must pay attention to the higher values of health losses of women with multiple roles.

Results support two hypotheses such that health losses of women with multiple roles were larger than those of men with multiple roles, and health losses of women with multiple roles can be reduced if the spouse shares the housework.

It is noted that one can reduce health losses of women with multiple roles to about 9.5% of the EHI if the spouses engage in frequent cleaning of the house. In contrast, the health losses of men with multiple roles can be reduced to around 12% of the EHI, if his wife engages in household cleaning at a high frequency. Employment and housework can substitute for each other, and this has positive effects on the health of women and men with multiple roles.

When excluding regional dummy variables, the estimated coefficients of SAPH were larger than that of the full model which included regional dummy variables, and the amount of evaluated health losses were estimated excessively as 13.2% and 10.9% for women with multiple roles and men with multiple roles, respectively (estimated coefficients are not shown). These results may indicate that regional dummy variables can explain the regional differences in gender roles on LS because the survey results [33] showed that husbands spent longer hours at work in a prefecture where the respondents strongly supported a traditional family structure, where the husband went out to work and the wife stayed at home. Thus, it would have been better if researchers used regional dummy variables while assessing the health losses of middle-aged workers.

I estimated generalized ordered probit (Goprobit) models, which allowed heterogeneity across both income and LS classes. As we moved to a higher LS category, a decrease in income gradient was found across LS categories for women with multiple roles (0.714, 0.642, 0.412) and men with multiple roles (0.771, 0.645, 0.461) (Tables 4 and 5). However, due to the remaining endogeneity where the generalized residuals were significant at the 5% level, no systematic relationships with regards to monetary compensations across LS categories in Goprobit models were found.

**Table 4:** Generalized ordered probit models in females: 2SRI approach.

Dependent Variables: LS	Female Workers with Multiple Roles				Female Workers with Few Roles			
	1st (lowest)	2nd	3rd	4th	1st (lowest)	2nd	3rd	4th
**Self-assessed poor health**	0.0192	-1.100***	-1.005***	-1.362***	-1.336	-1.386***	-1.414***	-0.251
	(1.010)	(0.402)	(0.324)	(0.464)	(1.038)	(0.348)	(0.292)	(0.329)
**Generalized residual**	-0.331	0.401	0.249	0.554**	0.494	0.409**	0.464***	-0.0296
	(0.624)	(0.253)	(0.187)	(0.250)	(0.588)	(0.205)	(0.170)	(0.191)
**Equivalent household income (logged)**	0.0203	0.714***	0.642***	0.412***	0.0389	0.148	0.443***	0.211***
	(0.602)	(0.123)	(0.0978)	(0.108)	(0.523)	(0.123)	(0.0802)	(0.0813)
**Age**	-0.164	-0.0195	0.0289	0.0262	-0.185*	0.0117	-0.0355	-0.0650***
	(0.136)	(0.0403)	(0.0296)	(0.0318)	(0.103)	(0.0391)	(0.0243)	(0.0240)
**Dummy variable for owning a house**	0.663	-0.0433	0.0610	0.194	-0.298	0.530***	0.267***	0.229**
	(0.452)	(0.168)	(0.110)	(0.122)	(0.353)	(0.121)	(0.0951)	(0.107)
**Dummy variable for income ratio to household income (1 = 50% or more, 0 other)**	0	0	5.141***	0.0697	-2.451**	0.156	0.0570	0.316
	(0)	(0)	(0.196)	(0.387)	(1.028)	(0.409)	(0.334)	(0.342)
**Frequency of cleaning by spouse**	0.406	0.175***	0.0978**	0.101**	0.0336	0.0701	-0.121**	0.0166
	(0.261)	(0.0602)	(0.0425)	(0.0462)	(0.277)	(0.0860)	(0.0567)	(0.0559)
***N***	1,007	1,007	1,007	1,007	1,202	1,202	1,202	1,202
**Average *N* per samples**	433	433	433	433	420	420	420	420

Note: Regional dummy variables such as Hokkaido, city size, frequency of preparation for meals by myself, frequent conversation with friends, and year dummy variable were included.

Standard errors in parentheses *** p<0.01, ** p<0.05, * p<0.1.

**Table 5:** Generalized ordered probit models in males: 2SRI approach.

Dependent variables: LS	Male workers with multiple roles				Male workers with few roles			
	1st (lowest)	2nd	3rd	4th	1st (lowest)	2nd	3rd	4^th^
**Self-assessed poor health**	0.257	-0.978**	-0.817**	-1.475***	21.35***	-2.119***	-2.756***	-0.675
	(1.288)	(0.412)	(0.333)	(0.484)	(1.035)	(0.686)	(0.563)	(0.688)
**Generalized residual**	-0.371	0.336	0.117	0.598**	-17.21***	1.061**	1.134***	0.228
	(0.717)	(0.262)	(0.196)	(0.264)	(0.771)	(0.442)	(0.324)	(0.387)
**Equivalent household income (logged)**	-0.148	0.771***	0.645***	0.461***	11.98***	0.357	0.293**	0.211
	(0.713)	(0.137)	(0.105)	(0.118)	(0.847)	(0.239)	(0.136)	(0.149)
**Age**	-0.172	-0.0148	0.0253	0.0306	1.501***	0.131*	0.0355	0.0262
	(0.146)	(0.0409)	(0.0303)	(0.0326)	(0.319)	(0.0750)	(0.0396)	(0.0433)
**Dummy variable for owning a house**	0.921*	-0.0252	0.0902	0.200	11.41***	0.457*	-0.114	0.335*
	(0.479)	(0.189)	(0.116)	(0.127)	(0.637)	(0.263)	(0.154)	(0.183)
**Dummy variable for income ratio to household income (1 = 50% or more, 0 other)**	0	0	4.911***	0.00236	4.294***	-1.071*	-0.768	-0.00335
	(0)	(0)	(0.191)	(0.387)	(1.585)	(0.613)	(0.506)	(0.704)
**Frequency of cleaning by spouse**	0.380	0.185***	0.105**	0.0984**	-1.784***	0.0992	0.0862	-0.00379
	(0.285)	(0.0642)	(0.0431)	(0.0471)	(0.162)	(0.0909)	(0.0562)	(0.0597)
***N***	957	957	957	957	476	476	476	476
**Average *N* per samples**	407	407	407	407	224	224	224	224

Note: Regional dummy variables such as Hokkaido, city size, frequency of preparation for meals by myself, frequent conversation with friends, and year dummy variable were included.

Standard errors in parentheses *** p<0.01, ** p<0.05, * p<0.1.

As theoretical analysis presented that the more restricting the time constraint is, the higher the individual’s valuation of time will be, and the more the individual will invest in health [34], and strategic interaction within the family may significantly influence health and health investment [35], it is recommended that a married couple share the total amount of time spent for non-market activities such as household cleaning.

Following the procedure of Mentzakis [36], we can use the average values of estimated significant coefficients and measure monetary compensation for the losses in well-being caused by poor health. The monetary values for health losses of women with multiple roles were 1.55 times (= 1.053/0.678) of the annual EHI, 22% larger than those of men with multiple roles. Among the 2-4th LS categories, health losses of women with multiple roles or men with multiple roles can be reduced if the spouse engages in household cleaning at high frequency.

The determinants of monetary compensation can be analyzed for health losses among working mothers in the highest LS category. For working mothers with few roles, the monetary value for health losses was zero (Table 4). Their LS can be, irrespective of their health status, explained by their age, household income level, and owning a house. A negative effect of age on LS was noted. Results suggest that the aging effect partially cancels out the effect of increased income on LS. In contrast, age, and owning a house were not significant among women with multiple roles. However, we cannot compare the monetary values for health losses among working mothers in the lowest LS category, because EHI was not significant in this category. WFC might occur in the lowest LS category if a mother’s or father’s participation in market work prevents them from fulfilling their family role. Future studies should include evaluations of a substantial WFC.

## Discussion

This study measured monetary values for health losses among middle-aged working persons with multiple roles. The random-effects ordered probit regression showed that the monetary values for health losses of women with multiple roles were 1.47 times of the EHI; larger than those of men with multiple roles. Because health losses of women with multiple roles can be reduced by around 9.5% of the EHI, if the spouses engage in frequent cleaning of the house, it is recommended that a married couple share the total amount of time spent for non-market activities such as household cleaning.

According to the 2016 Survey on Time Use by the Ministry of Internal Affairs and Communications in Japan, female caregivers in their 50s spent less time on child care than their 40s but spent more time on domestic work including shopping. In total, female caregivers in their 50s and 40s spent almost the same time on domestic work including caregiving. On the contrary, female non-caregivers in their 50s spent less time for domestic work than in their 40s. Thus, we can consider that men in their 50s whose wives engage in informal caregiving reconsider the allocation of work attributable to the attitudes toward gender roles.

Social norms in Japan expect married women to support their husband [37]. Therefore, middle-aged or elderly women have a less likelihood to benefit from marriage than men. Indeed, men with lifestyle diseases are more likely to be selected into marriage, while it is the opposite for women [38]. The findings of the study add to existing literature and can be compared globally to help understand where different countries stand. As discussed previously, the number of women with multiple roles has increased in Japan, but women still tend to assume primary responsibility for household duties, alongside their paid jobs. This holds true not only for Japan but also for other East-Asian countries where women are increasingly assuming multiple roles while still bearing the primary responsibility of traditional roles.

Taking health losses of women with multiple roles into consideration, middle-aged men should reconsider the allocation of work attributable to the attitudes toward gender roles. This conclusion derived from the study can help to reduce the gap between perceived health of women and increased roles among the different countries, and to reduce the disparity in the time spent for housework and childcare between married couples.

### Strengths and limitations

The results of generalized ordered probit models which allow heterogeneity across both income and LS classes showed that EHI was not significant in the lowest LS category. WFC might occur in the lowest LS category if a mother’s or father’s participation in market work prevents them from fulfilling their family role. Because this study used middle-aged cohort data, it is reasonable to assume that heterogeneous age effects are too small to be negligible. Indeed, no systematic heterogeneous age effects can be found among workers with multiple roles (Tables 3 and 4). However, female workers with few roles in the highest category where the generalized residual was not statistically significant had a negative age effect (Table 4). Those workers had an age effect on the SWB, but they did not have a significant effect of SAPH on SWB. Some female workers with few roles may have a heterogeneous effect of the variables across the SWB distribution. However, some results of this study are constrained to small sample size. A small sample size decreases statistical power which is necessary for avoiding Type II errors; therefore, we should consider that the results of male workers with few roles are less conclusive.

This study has some limitations. Almost 70% of workers with multiple roles hoped to be regular workers ten years later. This may indicate that employees are not randomly assigned to workplaces. Workers are not randomly assigned into high involvement management (HIM), and this may bias the estimates of HIM on employee well-being considerably [39]. However, due to no available data on employees’ work histories, HIM, and sickness absence histories, this study cannot examine this bias. Mothers’ personality traits on childcare which correlate with the other variables such as labor participation may bias the estimates. The lack of information about mothers’ personality traits is also a limitation of this study. Due to data limitations, eating behaviors such as frequent consumption of fruits and vegetables [40] were not taken into consideration in this study. The omitted variable of healthy eating may reduce the endogeneity bias between SAPH and LS among middle-aged workers with few roles. Although JLPS-M collected the data of childcare and the number of children for 3 years (2012–2014), it did not cover the sample period in this study. Then, these variables were not used as explanatory variables of estimation functions. In this study, engaging in housework at high frequency by oneself or the spouse was used as a proxy variable of childcare. Indeed, 93% of working mothers who frequently spent their time for housework participated in childcare during 2012–2014. In contrast, third variables may be omitted that can explain the change in the childcare environment. Because childcare environment, long working hours, and sharing the housework are mutually influenced, the decrease in the time spent for childcare may have positive effects on self-assessed health and LS. Because the 2007 JLPS-M survey provides the information about childcare environment, researchers should tackle the omitted variable problem described above in future studies.

## Conclusions

Taking health losses of women with multiple roles into consideration, middle-aged men should reconsider the allocation of work attributable to the attitudes toward gender roles. Although new legislation on work-style reforms was passed in 2018, including the introduction of legal upper limits on overtime hours with penalties, according to [41], the time spent on housework and child care by Japanese men was at the lowest level when compared globally. Japanese husbands with a child or children under six years spent 1 hour and 23 minutes on housework including child care per day. This was half the time spent by husbands in the UK. It is hoped that this legislation will contribute to reducing middle-aged men’s long working hours, and working wives’ WFC.

## Abbreviations

EHI: equivalent household income
IV: instrumental variable
JLPS-M: Japanese Life Course Panel Survey of the Middle-aged
LS: life satisfaction
MHI-5: the five-question Mental Health Inventory
SAPH: self-assessed poor health
SWB: subjective well-being
2SRI: two-stage residual inclusion
WFC: work-to-family conflict
